# Evaluating Antibiotic Treatment Guideline Adherence to Ongoing Antibiotic Stewardship in a Tertiary Care Setting: A Retrospective Observational Study

**DOI:** 10.1155/2024/6663119

**Published:** 2024-04-17

**Authors:** Suman Pant, Andrew Corwin, Prabhat Adhikari, Subhash Prasad Acharya, Upasana Acharya, Sashi Silwal, Pratima Dawadi, Anil Poudyal, Vibhu Paudyal, Adisak Bhumiratana

**Affiliations:** ^1^Government of Nepal, Nepal Health Research Council, Kathmandu, Nepal; ^2^Faculty of Public Health, Thammasat University, Rangsit Campus, Khlong Nueng, Pathum Thani 12121, Thailand; ^3^Department of Infection Prevention and Control, Grande International Hospital, Kathmandu, Nepal; ^4^Helen Keller International, Lalitpur, Nepal; ^5^Florence Nightingale Faculty of Nursing, Midwifery and Palliative Care, King's College London, London, UK; ^6^Thammasat University Research Unit in One Health and EcoHealth, Rangsit Campus, Khlong Nueng, Pathum Thani 12121, Thailand

## Abstract

Antimicrobial resistance (AMR) is widely regarded as an increasing threat to global public health. Antibiotic treatment guidelines have been increasingly recognized as an effective tool to guide appropriate prescriptions and help curtail antibiotic resistance. The present study aimed to assess physician's adherence to hospital antibiotic treatment guideline recommendations in Nepal and determine predictive variables with a significant association. This was a retrospective, monocentric observational review to investigate the adherence to endorsed guidelines using the medical records of adults admitted to the hospital with a diagnosis of urinary tract infection (UTI), pneumonia, or skin and soft tissue infection (SSTI) from January 2018 to December 2019. Of the 2,077 medical records that were reviewed (954 UTI, 754 pneumonia, and 369 SSTI), 354 (17%) met the study inclusion criteria, which included 87 UTI, 180 pneumonia, and 87 SSTI patients. Among eligible patients with antibiotic prescriptions, the following were adherent to guideline recommendations: 33 (37.9%) UTI, 78 (43.3%) pneumonia, and 23 (26.4%) SSTI. The overall extent of adherence to hospital antibiotic treatment guidelines for the use of antibiotics among adult inpatients diagnosed with these common infections was 37.9%. Patients who received ceftriaxone (OR = 2.09, 95% CI = 1.18–3.71, *p*=0.012) and levofloxacin (OR = 4.63, 95% CI = 1.30–16.53, *p*=0.018) had significantly higher adherence to treatment guidelines. This study revealed a low adherence rate despite the availability of updated guidelines for antibiotic prescriptions. The findings confer an urgent need to confront antibiotic prescription patterns in such tertiary care centers for tailored interventions to improve adherence to antibiotic guidelines.

## 1. Introduction

Antimicrobial resistance (AMR) is an emerging global health threat that has affected human health worldwide. The burden is significantly greater in low-income and middle-income countries (LMICs), including Nepal [1–3]. When antibiotics are no longer effective, the foundational elements and achievements of modern medicine and public health are undermined. The six leading pathogens responsible for deaths associated with AMR include *Escherichia coli*, followed by *Staphylococcus aureus*, *Klebsiella pneumoniae*, *Streptococcus pneumoniae*, *Acinetobacter baumannii*, and *Pseudomonas aeruginosa*) [4]. O'Neil estimated 700,000 deaths per year worldwide attributed to bacterial AMR with projections to increase to 10 million deaths by 2050 [5]. AMR contributes to deaths that surpass infectious and noncommunicable diseases combined; currently, resistance-related infections cause the premature death of one person every forty-five seconds. By 2050, the death toll is expected to increase to one person every three seconds unless improvements occur with the existing AMR surveillance at a national level [5]. AMR spread also poses a threat to achieving the Sustainable Development Goals for 2030, such as zero hunger, ending poverty, ensuring healthy lives, curtailing inequalities, and revitalizing sustainable developments. In the setting of climate change, AMR is expected to worsen [6]. AMR drives global catastrophic economic losses. The World Bank forecasted that the yearly global gross domestic product would decrease by 1.1% in the low-impact AMR scenario and by 3.8% in the high-impact scenario [7]. These losses parallel those provoked by the 2008-2009 global financial crisis, particularly in resource-limited settings. Overprescribing and/or misprescription of antibiotics are the major contributors to AMR. Sequentially, this augments the risk of adverse effects and hospital readmissions for patients. In addition, it leads to increased healthcare spending due to drug-resistant infections [5, 8–10]. Globally, the overall compliance to antibiotic treatment guidelines in the hospital was 77.4%. The proportion was less than seventy percent in West and Central Asia, Latin America, and Africa [11].

The World Health Organization (WHO), in collaboration with various national, international, and professional organizations, introduced antimicrobial stewardship programs (ASPs). These programs are designed as comprehensive healthcare strategies to promote, improve, monitor, and evaluate the appropriate use of antibiotics in humans and animals with the goal of curbing the emergence and spread of AMR [12–15]. In Nepal, the Ministry of Health and Population and the Department of Health Services have endorsed the National Antibiotic Treatment Guideline 2014 and the National Antimicrobial Resistance Containment Action Plan Nepal 2016, respectively. The ASP, adopted through policies and regulatory measures, serves as a regulatory approach. Its purpose is to optimize the prudent use of antimicrobials, enhance patient outcomes, and reduces costly healthcare-associated infections (HCAIs) such as pneumonia, urinary tract infections (UTIs), and skin and soft tissue infections (SSTIs) nationwide [15–20]. Nevertheless, the ASP implementation necessitates information on prior patterns of antibiotic usage for treating patients admitted to the hospital with common HCAIs and the quality of prescribing practices at national and subnational levels.

This study aimed to assess the extent of empiric antibiotic treatment guideline adherence to common infections requiring an antibiotic prescription and determine the factors associated with adherence in a tertiary care center with an established ASP in Nepal.

## 2. Methods

### 2.1. Study Design and Setting

We conducted a single-center, retrospective analysis of the medical records of the patients admitted with pneumonia, urinary tract infections, and skin and soft tissue infections between 1 January 2018 and 31 December 2019. This study involved systemic and ongoing collection of baseline data and information regarding empiric antibiotic usage, adherence to hospital treatment guidelines, and quality of antibiotic prescribing based on WHO-recommended evaluation algorithms. It was conducted at Grande International Hospital, Kathmandu, Nepal, a 200-bed multispecialty national referral tertiary care center with ongoing ASP implementation at its specialist units (e.g., hematology, burns, renal, and oncology) and specialized intensive care units (e.g., medical, dental surgical, neurosurgical cardiothoracic, and vascular surgery).

### 2.2. Study Population and Selection Criteria

**Figure 1:**
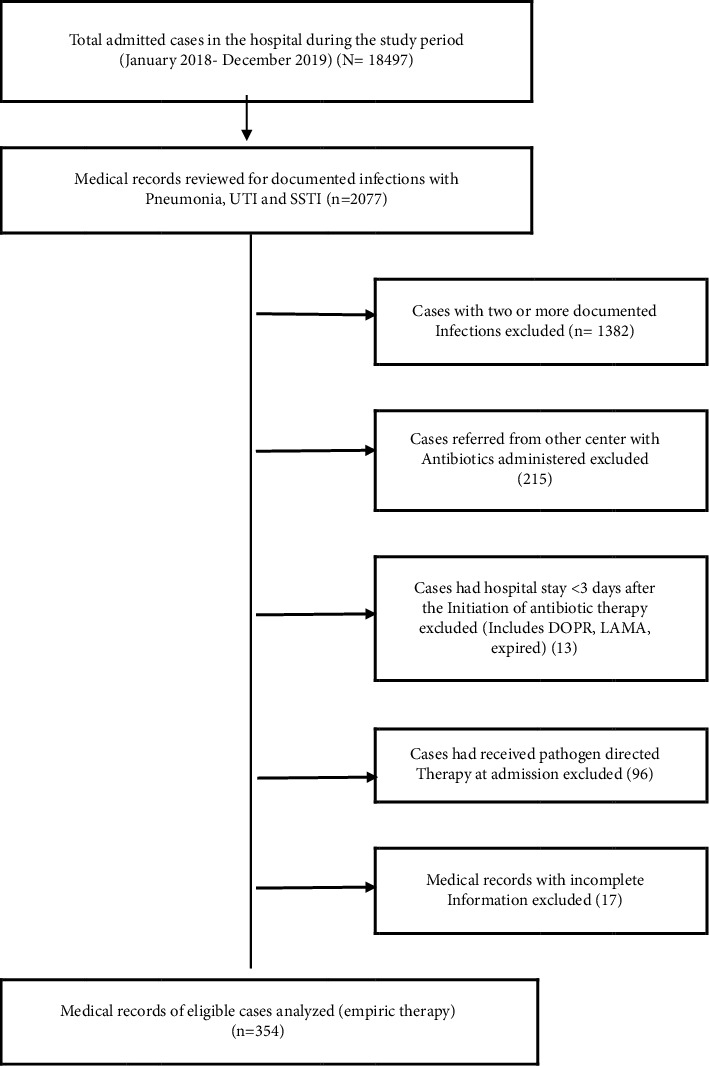
A schema for the selection of the admitted patients with the eligibility in this study.

Based on the hospital information system and the retrieval system, this study reviewed the medical records of 2,077 patients aged 15 years and older who were admitted during the study and were diagnosed as ICD-10 code cases with pneumonia (*n* = 954), UTI (*n* = 754), and SSTI (*n* = 369) (Figure 1). The 354 hospital-admitted patients who were eligible for a complete medical record review were treated with empiric therapy and were selected from the respective inpatient departments. Using the validated clinical record form, the eligible records of the study patients with specific conditions as classified by the ICD-10 were meticulously screened and selected through onsite manual data collection from paper-based medical records in the absence of both selection and information biases. This cohort of hospitalized patients receiving empiric antibiotic regimens was subjected to further analysis of adherence to treatment guidelines. As the hospital's guidelines were tailored for single-infection treatment, individual inpatients with two or more documented infections were excluded, as multiple infections could complicate adherence assessment. In addition, those who were referred from other centers, and more likely, on antibiotics were excluded in order to follow the hospital's initial prescribing practices. Those who had a hospital stay of less than three days' postantibiotic initiations were also excluded because their duration of hospitalization was insufficient to meet a complete antibiotic therapy and any deescalation based on susceptibility testing. Pregnant women were excluded from the study due to the lack of hospital guidelines that can provide specific information on empiric regimens for infections requiring antibiotic therapy during pregnancy or safe drug use. Those receiving prophylactic antibiotic treatments were also not considered. Those who had incomplete medical records or lacked adequate documentation of antibiotic prescriptions, dosages, and treatment durations were also excluded to ensure data accuracy and reliability. These methods of selection were reliable enough to warrant a homogenous cohort of the study patients and a reliable analysis of guideline adherence and prescribing patterns within the hospital's antimicrobial stewardship program.

### 2.3. Data Collection Methods and Patient Data Sources

The validated paper-based clinical record form (CRF) [21] with admissible 28 items having acceptable item objective congruence indices was used to extract the specific patient data, which were based on WHO indicators [22], from the electronic medical records at the Medical Record Department with permission from the hospital and the responsible medical directors. Patient data on unique hospital numbers archived by using the ICD-10 code, cases with pneumonia, UTI, and SSTI (Supplementary Table 1) included demographic data, clinical diagnosis, antibiotic prescription data, antibiotic class, route of administration, duration of therapy, and antimicrobial sensitivity test results. Along with the index disease under study, the presence of any additional comorbid condition in the patients was also recorded and considered. These comorbid conditions include chronic or acute disease (but not infectious diseases) or other medical conditions in the same inpatients. This is important as comorbidities can significantly influence outcomes of the primary conditions, affect treatment choices, and impact the overall prognosis of the patient. As for the medical records review, the documents required included admission notes, medical and nursing charts, medication charts, and discharge summaries. They were verified, if not properly documented, by asking any treating physician/medical doctor. Their verbal or written informed consents where appropriate, were obtained. Given the secondary review and claims of data from the hospital medical records, informed consent of the study participants was not applicable.

### 2.4. Assessment of Adherence to Therapeutic Guidelines and Variables

The analysis of the documented prescriptions was based on the established antibiotic treatment guidelines of the hospital for UTI, pneumonia, and SSTI (Supplementary Table 2). International standard antibiotic treatment guidelines [23–28] were adopted to assess the empiric treatment regimens for other SSTIs.

Adherence was defined as the physician's antibiotic prescribing practice that aligned with guidelines for empiric antibiotic selection, dosage, and duration of therapy. Nonadherence to the standard antibiotic treatment guideline was referred to as a prescription of inappropriate choice (not indicated antibiotics in the guideline) or failure to have full concordance with the other components of antibiotic prescription: dosage and duration [29]. A complete review of the medical charts was performed for guideline nonadherent treatment. The assessment and determination of the medical charts were carried out by a team of six assessors, comprising the PI and five other investigators with expertise in various medical fields, including intensivists, infectious disease physicians, clinical pharmacists, microbiologists, and research nurses. Any deviations that were referred to as incomplete components of the empiric antibiotic regimen (selection, dosage, and duration) were initially evaluated to see whether the physician had attempted to treat based on the hospital antibiogram trends and/or patient-specific factors. Moreover, the patient-specific factors, which were indicative of treatment by physicians, included the history of allergies, recent antibiotic exposure, and suspected coinfection in the presence or absence of accompanying clinical outcomes or infections [30]. These indices were deemed necessary for further assessment by two infectious disease specialists.

A framework for assessing adherence to therapeutic guidelines was performed using several variables. The dependent variables were treatment via guidelines, guideline nonadherent treatment, and prescribing errors. Incorrect selection of antibiotics, incorrect dosage, and incorrect duration alignment with therapeutic guideline recommendations were also included. The incorrect dosage was further subdivided into insufficient and excessive dosage, defined as a dosage prescribed less or more in comparison with therapeutic guideline recommendations. Similarly, the incorrect duration was further categorized into insufficient and excessive duration. The independent variables comprised the following variables: unit/department, gender, age, presence of comorbidities, comorbidities, infectious diseases, antibiotic (drug) allergy, and drug sensitivity test and empiric antibiotics, similar to other studies [31–36].

### 2.5. Data Analysis and Statistical Tests

Descriptive statistics such as frequencies and percentages were used to describe nominal and dichotomous variables, as the mean and standard deviation were used for continuous variables. The two-group comparison of patient demographics and other characteristics was made between “guideline adherent treatment” and “guideline nonadherent treatment.” Univariate and multivariate logistic regression analyses, where stepwise regression was used in selecting predictive variables at *p* < 0.1 in the final fitted model, were performed to determine a statistically significant relationship (*p* < 0.05) between the dependent and independent variables. Crude odds ratio (OR), or adjusted OR with 95% confidence intervals (CIs), was calculated to describe the pairwise association between the guideline adherent treatment (or guideline nonadherent treatment) and the independent variables.

## 3. Results

### 3.1. General Description of Inpatients

Of the 2,077 inpatients diagnosed as ICD-10 code case classification: 954 UTI, 754 pneumonia, and 369 SSTI, there were 354 (17.0%) patients admitted with complete medical record review eligible for empiric therapy. About 57.1% were males with an average age of 55 years. There were variable proportions of 354 inpatients with underlying diseases or comorbidities: 78.0% with administered antibiotics at the time of discharge, 69.8% with the correct selection of empiric antibiotics, and 37.9% of the inpatients receiving empiric therapy as per the guideline's recommendations (Table 1).

Moreover, the resulting diagnostic tests were likely to be differentiated for inpatients with UTI, pneumonia, and SSTI. Urine samples were attained and reported for antimicrobial susceptibility testing (AST) for 97.7% of the inpatients empirically treated for UTI. Similarly, biological specimens (blood, sputum, and endotracheal aspirate) were collected for AST among 81.7% of inpatients with pneumonia. For SSTI, clinical samples (pus, wound aspirate, and necrotic tissue) were collected in three-fourths (74.7%) of inpatients only. Culture results were positive for 129 (36.4%) of the clinical samples analyzed. Clinical samples were not sent for culture tests for 57 (16.1%) inpatients. The imaging studies for these infections performed at the start of the antibiotic treatment elicited high-to-low rankings with proportions of pneumonia (95.0%), SSTI (34.5%), and UTI (28.7%), respectively. The signs of infection in the imaging studies were evident amongst 217 (61.3%) of the inpatients. For 98% of the patients empirically diagnosed with UTI, clinical specimens were collected either on the day of therapy or before day 1, while this was only 68% with pneumonia and 45% with SSTI patients (Table 1).

### 3.2. Factors Associated with Adherence to Standard Treatment Guidelines

Of the 354 inpatients including 180 with pneumonia and the same 87 UTI and SSTI patients, there were 134 (37.9%) with guideline adherent treatment and 220 (62.1%) with guideline nonadherent treatment (Table 2). In other words, there were 33 (37.9%) UTI patients, 78 (43.3%) pneumonia patients, and 23 (26.4%) SSTI patients who adhered to the treatment guidelines, respectively.

Based on univariate logistic regression analysis (Table 2), significant associations were evident with variables such as ICU (OR = 0.44, 95% CI = 0.22–0.90, *p*=0.020), inpatients aged >65 years (OR = 0.53, 95% CI = 0.31–0.91, *p*=0.021), and the presence of comorbidities (OR = 0.44, 95% CI = 0.26–0.75, *p*=0.003). Regarding the empiric antibiotics, adherence to treatment guidelines had strongly significant associations with increased use of empiric antibiotics such as ceftriaxone (OR = 2.11, 95% CI = 1.24–3.60, *p*=0.006) and levofloxacin (OR = 4.37, 95% CI = 1.27–14.99, *p*=0.019), but seemed to have significant association with decreased use of azithromycin (OR = 0.47, 95% CI = 0.30–0.74, *p*=0.001).

In the multivariate logistic regression model, the inpatients receiving ceftriaxone (AOR = 2.09, 95% CI = 1.18–3.71, *p*=0.012) and levofloxacin (AOR = 4.63, 95% CI = 1.30–16.53, *p*=0.018) were significantly associated with the adherence to treatment guidelines. Regarding this, some confounding factors for such regression analysis included unit/department, patient age group, and presence of comorbidities.

## 4. Discussion

This study aimed to investigate the adherence of physicians to relevant standard treatment guidelines for the diagnosis and treatment of common bacterial infections at a tertiary referral hospital in Nepal. The findings suggest that the overall adherence to standard treatment guidelines for antibiotic prescribing in terms of drug selection, dosage, and duration in the studied hospital was suboptimal at only 37.9%. The observation of the suboptimal-level adherence rate was congruent with that observed by previous studies in other European countries [37, 38], but contrasted with some studies reporting higher levels of antimicrobial treatment guideline adherence rates: 53% observed in Denmark [39], 56% in the Netherlands [40], 57% in the Republic of Korea [41], and 59% in Canada [42]. In addition, Ekman et al. [43] reported high adherence to treatment guidelines in Nepal in infants.

The finding of the univariate analysis in this study indicated that the standard treatment guidelines were likely to be adhered to when prescribing antibiotics for inpatients aged >65 years. This observation did not rule out the possibility that the increasing age of the inpatients had an increased association with empirical antibiotics and comorbidities. Notably, adherence to treatment guidelines was strongly attributable to currently available antibiotics used in the empirical treatment in the studied hospital. The empiric antibiotics were azithromycin, ceftriaxone, and levofloxacin. Azithromycin was taken into consideration as the most frequently used prescribed antibiotic, followed by piperacillin/tazobactam. In a similar fashion, several studies undertaken in different healthcare settings in Nepal demonstrated that azithromycin is the most frequently prescribed antibiotic in outpatient departments [44–46], probably due to its high availability, safety, and affordability. Regarding ceftriaxone and levofloxacin, the significant association of adherence to treatment guidelines was in alignment with previous findings observed in Cape Town Metro District, South Africa [34]. Interestingly, levofloxacin and ceftriaxone were commonly prescribed, with an average of 4.7 and 2.2 times, respectively, in adherence to the hospital treatment guidelines. Taken together, the discrepancy in adherence to treatment guidelines might be due to poor implementation of prescribing practices for hospitalized inpatients and inappropriate and excessive use of antibiotics, as supported by the emergence and spread of AMR in healthcare settings. Further large-scale research is needed to investigate antimicrobial prescribing in hospital settings, including the identification of risk factors associated with inappropriate prescribing. This will enable the development of targeted intervention strategies to effectively address the impact of AMR in Nepal.

The study is the first of its kind in the context of Nepal in assessing the extent of guideline adherence of the prescribing physicians in the antibiotic treatment and prescription pattern in the adult inpatients. In addition, this study describes the entire process of antibiotic stewardship implemented in the inpatient department of the hospital. The detailed insight into the process may serve as a valuable reference for other healthcare facilities planning to implement similar antibiotic stewardship programs and facilitates knowledge sharing and the adoption of best practices across the healthcare sector. However, this was a single-center retrospective observational study. Thus, the findings may have limited generalizability.

This study also acknowledges the challenge of drawing direct comparisons with studies focusing on community antibiotic use or those addressing different conditions in hospital settings. While we endeavored to identify other hospital-based studies evaluating the same infections, our search did not yield identical studies for a direct comparison. Thus, this study underscores a thorough analysis of the inherent differences between the settings and disease conditions and those of the existing literature. This analysis not only contextualizes our findings within the broader landscape of antibiotic stewardship research but also emphasizes the innovative aspect of the study in addressing a significant gap in the literature.

## 5. Conclusion

This study demonstrated a suboptimal antibiotic adherence rate for patients admitted for UTI, pneumonia, and SSTI, despite updated treatment guidelines for antibiotic prescriptions for common infections. Results represent an opportunity for hospitals in Nepal and other low-income and middle-income countries to improve evidence-based antibiotic practices and implementation processes to reduce unnecessary costs, complications, and AMR. The results are especially imperative for older patients hospitalized with comorbidities as well as an aging population in years to come. Urgently addressing antibiotic stewardship in hospitals now could improve patient-centered health outcomes, particularly with co-occurring crises such as climate change, and ensure the best treatment availability and efficacy for patients in resource-constrained settings.

## Figures and Tables

**Table 1:** Demographic and Clinical Profiling of the 354 eligible inpatients in this study.

Variables	No. of inpatients (%)			
	UTI	Pneumonia	SSTI	Total
	(*n* = 87)	(*n* = 180)	(*n* = 87)	(*N* = 354)
*Gender*				
Male	43 (49.4)	100 (55.6)	59 (67.8)	202 (57.1)
Female	44 (50.6)	80 (44.4)	28 (32.2)	152 (42.9)
*Age groups (years): mean* *±* *SD 55.4* *±* *19.6*				
Below 45	32 (36.8)	29 (16.1)	49 (56.3)	110 (31.0)
46–65	34 (39.1)	61 (33.9)	27 (31.0)	122 (34.5)
Above 65	21 (24.1)	90 (50.0)	11 (12.7)	122 (34.5)
*Presence of comorbidities*				
Yes	70 (80.5)	155 (86.1)	29 (33.3)	254 (71.7)
No	16 (18.4)	25 (13.9)	52 (59.7)	93 (26.3)
Unknown	1 (1.1)	0 (0.0)	6 (7.0)	7 (2.0)
*Chronic obstructive pulmonary disease (COPD)*				
Yes	4 (4.6)	52 (28.9)	2 (2.3)	58 (16.4)
No	83 (95.4)	128 (71.1)	85 (97.7)	296 (83.6)
*Hypertension*				
Yes	38 (43.7)	103 (57.2)	15 (17.2)	156 (44.1)
No	49 (56.3)	77 (42.8)	72 (82.8)	198 (55.9)
*Diabetes mellitus*				
Yes	34 (39.1)	56 (31.1)	13 (14.9)	103 (29.1)
No	53 (60.9)	124 (68.9)	74 (85.1)	251 (70.9)
*Coronary artery disease*				
Yes	3 (3.4)	40 (22.2)	1 (1.1)	44 (12.4)
No	84 (96.6)	140 (77.8)	86 (98.9)	310 (87.6)
*Specimen collected*				
Yes	85 (97.7)	147 (81.7)	65 (74.7)	297 (83.9)
No	2 (2.3)	33 (18.3)	22 (25.3)	57 (16.1)
*Microbiology culture*				
Positive	40 (46.0)	54 (30.0)	35 (40.2)	129 (36.4)
Negative	45 (51.7)	93 (51.7)	30 (34.5)	168 (47.5)
No culture	2 (2.3)	33 (18.3)	22 (25.3)	57 (16.1)
*Imaging study at the start of antibiotics*				
Yes	25 (28.7)	171 (95.0)	30 (34.5)	226 (63.8)
No	62 (71.3)	9 (5.0)	57 (65.5)	128 (36.2)
*Sign of infection on imaging studies*				
Yes	20 (23.0)	167 (92.8)	30 (34.5)	217 (61.3)
No	5 (5.8)	4 (2.2)	0 (0.0)	9 (2.5)
No study	62 (71.2)	9 (5.0)	57 (65.5)	128 (36.2)
Clinical specimen collection time				
On the day of therapy/before day 1	85 (97.7)	122 (67.8)	39 (44.8)	246 (69.5)
After the start of empirical therapy	0 (0)	25 (13.9)	26 (29.9)	51 (14.4)
No culture	2 (2.3)	33 (18.3)	22 (25.3)	57 (16.1)
*Antibiotics prescribed at the time of discharge*				
Yes	72 (82.8)	133 (73.9)	71 (81.6)	276 (78.0)
No	15 (17.2)	47 (26.1)	16 (18.4)	78 (22.0)
*Correct selection of empiric antibiotics*				
Yes	66 (75.9)	137 (76.1)	44 (50.6)	247 (69.8)
No	21 (24.1)	43 (23.9)	43 (49.4)	107 (30.2)
*Correct dosage of empiric antibiotics*				
Yes	64 (73.6)	136 (75.6)	44 (50.6)	244 (68.9)
No	23 (26.4)	44 (24.4)	43 (49.4)	110 (31.1)
*Correct duration of empiric antibiotics*				
Yes	33 (37.9)	78 (43.3)	23 (26.4)	134 (37.9)
No	54 (62.1)	102 (56.7)	64 (73.6)	220 (62.1)

Yes, having the status of the current condition of the disease or biological sample collected or imaging study performed at the start of antibiotics or sign of infection imaging studies or receiving antibiotic treatment; No, neither having the status of the current condition of the disease nor biological sample collected nor imaging study performed at the start of antibiotics nor sign of infection imaging studies nor receiving antibiotic treatment.

**Table 2:** Univariate and multivariate logistic regression analyses results determining the predictive variables for guideline adherent treatment.

Variables	No. (%) of adherence	No. (%) of nonadherence	Univariate analysis		Multivariate analysis^a^	
			OR (95% CI)	*P* value	AOR (95% CI)	*P* value
*Unit/department (n* *=* *354)*						
General medical ward	56 (38.6)	89 (61.4)	Ref	0.048^*∗*^	Ref	
Surgical ward	11 (27.5)	29 (72.5)	1.65 (0.76–3.58)	0.198	1.19 (0.47–3.01)	0.707
Orthopedics	12 (29.3)	29 (70.7)	1.52 (0.71–3.22)	0.274	1.35 (0.53–3.46)	0.531
Urology	1 (6.7)	5 (83.3)	3.14 (0.35–27.63)	0.301	1.93 (0.19–19.13)	0.574
ICU	24 (58.5)	17 (41.5)	0.44 (0.22–0.90)	0.020^*∗*^	0.65 (0.31–1.37)	0.256
Others	30 (37.0)	51 (63.0)	1.07 (0.61–1.87)	0.814	1.29 (0.69–2.43)	0.429
*Patient age group (n* *=* *354)*						
Below 45 years	35 (31.8)	75 (68.2)	Ref	0.042^*∗*^	Ref	
45–65 years	42 (34.4)	80 (65.6)	0.89 (0.51–1.54)	0.674	1.09 (0.58–2.02)	0.796
>65 years	57 (46.7)	65 (53.3)	0.53 (0.31–0.91)	0.021^*∗*^	0.86 (0.45–1.64)	0.669
*Gender (n* *=* *354)*						
Male	82 (40.6)	120 (59.4)	Ref	0.008^*∗*^	—	—
Female	52 (34.2)	100 (65.8)	1.31 (0.85–2.04)	0.221	—	—
*Presence of comorbidities (n* *=* *347)*^*b*^						
No	23 (24.7)	70 (75.3)	Ref	0.011^*∗*^	Ref	
Yes	108 (42.5)	146 (57.5)	0.44 (0.26–0.75)	0.003^*∗*^	0.52 (0.27–1.00)	0.050
*Infectious disease diagnosed (n* *=* *354)*						
UTI	33 (37.9)	54 (62.1)	Ref	0.030	—	—
Pneumonia	78 (43.3)	102 (56.7)	0.80 (0.47–1.35)	0.402	—	—
SSTI	23 (26.4)	64 (73.6)	1.70 (0.89–3.24)	0.106	—	—
Empiric antibiotics						
Azithromycin	67 (48.6)	71 (51.4)	0.47 (0.30–0.74)	0.001^*∗*^	0.73 (0.42–1.26)	0.255
Piperacillin/tazobactam	54 (44.6)	67 (55.4)	0.65 (0.41–1.02)	0.059	—	—
Ceftriaxone	23 (25.6)	67 (74.4)	2.11 (1.24–3.60)	0.006^*∗*^	2.09 (1.18–3.71)	0.012∗
Amoxicillin/clavulanic acid	28 (38.4)	45 (61.6)	0.97 (0.57–1.65)	0.921	—	—
Meropenem	15 (40.5)	22 (59.5)	0.88 (0.44–1.76)	0.722	—	—
Cefazolin	10 (40.0)	15 (60.0)	1.10 (0.48–2.53)	0.818	—	—
Levofloxacin	3 (13.0)	20 (87.0)	4.37 (1.27–14.99)	0.019^*∗*^	4.63 (1.30–16.53)	0.018^*∗*^

^*∗*^Significance at *p* < 0.05. AOR = adjusted odds ratio; — = not significantly associated. ^a^Only included the variables significant at *p* “level 0.1.” ^b^excluding unknown cases.

## Data Availability

The data used to support the findings of this study are available from the corresponding authors upon request.

## Supplementary Materials

Supplementary Materials Supplementary Table 1: infectious disease diagnosis of patients and ICD-10 categories. Supplementary Table 2: hospital's antibiotic treatment guideline.

## References

[1] Laxminarayan R., Van Boeckel T., Frost I. (2020). The lancet infectious diseases commission on antimicrobial resistance: 6 years later. *The Lancet Infectious Diseases*.

[2] Pokharel S., Raut S., Adhikari B. (2019). Tackling antimicrobial resistance in low-income and middle-income countries. *BMJ Global Health*.

[3] Rose A. N., Baggs J., Wolford H. (2021). Trends in antibiotic use in United States hospitals during the coronavirus disease 2019 pandemic. *Open Forum Infectious Diseases*.

[4] Murray C. J., Ikuta K. S., Sharara F. (2022). Global burden of bacterial antimicrobial resistance in 2019: a systematic analysis. *The Lancet*.

[5] O’Neill J. (2014). Antimicrobial resistance: tackling a crisis for the health and wealth of nations- the Review on Antimicrobial Resistance. https://amr-review.org/sites/default/files/AMRReviewPaper-Tacklingacrisisforthehealthandwealthofnations.pdf.

[6] Magnano San Lio R., Favara G., Maugeri A., Barchitta M., Agodi A. (2023). How antimicrobial resistance is linked to climate change: an overview of two intertwined global challenges. *International Journal of Environmental Research and Public Health*.

[7] Jonas O. B., Irwin A., Berthe F. C. J., Le Gall F. G., Marquez P. V. (2017). Drug-resistant Infections: a threat to our economic future (Vol 2). https://documents.worldbank.org/en/publication/documents-reports/documentdetail/323311493396993758/final-report.

[8] Llor C., Bjerrum L. (2014). Antimicrobial resistance: risk associated with antibiotic overuse and initiatives to reduce the problem. *Therapeutic Advances in Drug Safety*.

[9] World Health Organization (1970). *Monitoring and Evaluation of the Global Action Plan on Antimicrobial Resistance: Framework and Recommended Indicators*.

[10] Sifri Z., Chokshi A., Cennimo D., Horng H. (2019). Global contributors to antibiotic resistance. *Journal of Global Infectious Diseases*.

[11] Versporten A., Zarb P., Caniaux I. (2018). Antimicrobial consumption and resistance in adult hospital inpatients in 53 countries: results of an internet-based Global Point Prevalence Survey. *Lancet Global Health*.

[12] World Health Organization (2015). *Global Action Plan on Antimicrobial Resistance*.

[13] Ahmad R., Zhu N. J., Leather A. J., Holmes A., Ferlie E. (2019). Strengthening strategic management approaches to address antimicrobial resistance in Global Human Health: a scoping review. *BMJ Global Health*.

[14] Majumder M. A., Rahman S., Cohall D. (2020). Antimicrobial stewardship: fighting antimicrobial resistance and protecting global public health. *Infection and Drug Resistance*.

[15] Rahman M. M., Alam Tumpa M. A., Zehravi M. (2022). An overview of antimicrobial stewardship optimization: the use of antibiotics in humans and animals to prevent resistance. *Antibiotics*.

[16] Yoon Y. K., Kwon K. T., Jeong S. J. (2021). Guidelines on implementing antimicrobial stewardship programs in Korea. *Infection & Chemotherapy*.

[17] Nauriyal V., Rai S. M., Joshi R. D. (2020). Evaluation of an antimicrobial stewardship program for wound and burn care in three hospitals in Nepal. *Antibiotics*.

[18] Davey P., Marwick C. A., Scott C. L. (2017). Interventions to improve antibiotic prescribing practices for hospital inpatients. *Cochrane Database of Systematic Reviews*.

[19] Acharya K. P., Wilson R. T. (2019). Antimicrobial resistance in Nepal. *Frontiers of Medicine*.

[20] Dahal R. H., Chaudhary D. K. (2018). Microbial infections and antimicrobial resistance in Nepal: current trends and recommendations. *The Open Microbiology Journal*.

[21] Fröhlich D., Bittersohl C., Schroeder K. (2019). Reliability of paper-based routine documentation in psychiatric inpatient care and recommendations for further improvement. *Frontiers in Psychiatry*.

[22] World Health Organization (2012). *How to Investigate Antimicrobial Use in Hospitals: Selected Indicators*.

[23] Stevens D. L., Bisno A. L., Chambers H. F. (2014). Executive summary: practice guidelines for the diagnosis and management of skin and soft tissue infections: 2014 update by the Infectious Diseases Society of America. *Clinical Infectious Diseases*.

[24] Kwak Y. G., Choi S.-H., Kim T. (2017). Clinical guidelines for the antibiotic treatment for community-acquired skin and soft tissue infection. *Infection & Chemotherapy*.

[25] Indian Council of Medical Research (2019). Treatment guidelines for antimicrobial use in common syndromes. https://main.icmr.nic.in/sites/default/files/guidelines/Treatment_Guidelines_2019_Final.pdf.

[26] American College of Clinical Pharmacy (2015). Skin and soft tissue infections [Internet]. https://www.accp.com/docs/bookstore/psap/2015B1.SampleChapter.pdf.ACCA.

[27] Kalil A. C., Metersky M. L., Klompas M. (2016). Management of adults with hospital-acquired and ventilator-associated pneumonia: 2016 clinical practice guidelines by the Infectious Diseases Society of America and the American Thoracic Society. *Clinical Infectious Diseases*.

[28] American Thoracic Society (2005). Guidelines for the management of adults with hospital-acquired, ventilator-associated, and healthcare-associated pneumonia. *American Journal of Respiratory and Critical Care Medicine*.

[29] Gyssens I. C., Van Den Broek P. J., Kullberg B.-J., Hekster Y. A., Van Der Meer J. W. (1992). Optimizing antimicrobial therapy. A method for antimicrobial drug me evaluation. *Journal of Antimicrobial Chemotherapy*.

[30] Thompson R. L., Wright A. J. (1998). General principles of antimicrobial therapy. *Mayo Clinic Proceedings*.

[31] Aly N. Y., Omar A. A., Badawy D. A., Al-Mousa H. H., Sadek A. A. (2012). Audit of physicians’ adherence to the antibiotic policy guidelines in Kuwait. *Medical Principles and Practice*.

[32] Radošević Quadranti N., Popović B., Škrobonja I., Skočibušić N., Vlahović-Palčevski V. (2014). Assessment of adherence to printed guidelines for antimicrobial drug use in a University Hospital. *European Journal of Hospital Pharmacy*.

[33] Grenet J., Davido B., Bouchand F. (2016). Evaluating antibiotic therapies prescribed to adult patients in the emergency department. *Medecine et Maladies Infectieuses*.

[34] Gasson J., Blockman M., Willems B. (2018). Antibiotic prescribing practice and adherence to guidelines in primary care in the cape town metro district, South Africa. *South African Medical Journal*.

[35] Pouwels K. B., Hopkins S., Llewelyn M. J., Walker A. S., McNulty C. A., Robotham J. V. (2019). Duration of antibiotic treatment for common infections in English primary care: cross sectional analysis and comparison with guidelines. *BMJ*.

[36] Liu P., Ohl C., Johnson J., Williamson J., Beardsley J., Luther V. (2016). Frequency of empiric antibiotic de-escalation in an acute care hospital with an established Antimicrobial Stewardship Program. *BMC Infectious Diseases*.

[37] Rossio R., Franchi C., Ardoino I. (2015). Adherence to antibiotic treatment guidelines and outcomes in the hospitalized elderly with different types of pneumonia. *European Journal of Internal Medicine*.

[38] Philips H., Huibers L., Holm Hansen E. (2014). Guidelines adherence to lower urinary tract infection treatment in out-of-hours primary care in European countries. *Quality in Primary Care*.

[39] Hagen T. L., Hertz M. A., Uhrin G. B., Dalager-Pedersen M., Schønheyder H. C., Nielsen H. (2017). Adherence to local antimicrobial guidelines for initial treatment of community-acquired infections. *Danish Medical Journal*.

[40] Schuttevaer R., Brink A., Alsma J. (2020). Non-adherence to antimicrobial guidelines in patients with bloodstream infection visiting the emergency department. *European Journal of Internal Medicine*.

[41] Kang S. H., Jo Y. H., Lee J. H., Jang D.-H., Kim Y. J., Park I. (2021). Antibiotic prescription consistent with guidelines in emergency department is associated with 30-day survival in severe community-acquired pneumonia. *BMC Emergency Medicine*.

[42] Fortin É, Deceuninck G., Sirois C. (2022). Chronic diseases and compliance with provincial guidelines for outpatient antibiotic prescription in cases of otitis media and respiratory infections: a population-based study of Linked Data in Quebec, Canada, 2010–2017. *CMAJ Open*.

[43] Ekman B., Paudel P., Basnet O., Kc A., Wrammert J. (2020). Adherence to World Health Organisation guidelines for treatment of early onset neonatal sepsis in low-income settings; a cohort study in Nepal. *BMC Infectious Diseases*.

[44] Pokharel S., Basnyat B., Arjyal A. (2017). Co-trimoxazole versus azithromycin for the treatment of undifferentiated febrile illness in Nepal: study protocol for a randomized controlled trial. *Trials*.

[45] Van Boeckel T. P., Gandra S., Ashok A. (2014). Global Antibiotic Consumption 2000 to 2010: an analysis of national pharmaceutical sales data. *The Lancet Infectious Diseases*.

[46] Giri A., Karkey A., Dangol S. (2020). Trimethoprim-sulfamethoxazole versus azithromycin for the treatment of undifferentiated febrile illness in Nepal: a double-blind, randomized, placebo-controlled trial. *Clinical Infectious Diseases*.

