# Supplementary material for: Evaluating Antibiotic Treatment Guideline Adherence to Ongoing Antibiotic Stewardship in a Tertiary Care Setting: A Retrospective Observational Study
Source: Can J Infect Dis Med Microbiol. 2024 Apr 17;2024:6663119. doi: 10.1155/2024/6663119 (PMC11042908; doi:10.1155/2024/6663119)
Supplement: Supplementary Materials — Supplementary Table 1: infectious disease diagnosis of patients and ICD-10 categories. Supplementary Table 2: hospital's antibiotic treatment guideline. [file 6663119.f1.zip › Supplemenatry_Table1_v1.1_14.2.2024.docx]

**Supplementary Table 1:** Infectious disease diagnosis of patients and ICD-10 categories

| **Categories** | **Indications for treatment** |
| --- | --- |
| Urinary Tract Infections (UTI) | N10, N13.2, N15.1, N30.0, N30.01, N30.8, N41.0, N41.2, N41.3, T83.511A, T83.592A |
| Pneumonia | J13, J14, J15.0, J15.1, J15.2, J15.3, J15.4, J15.5, J15.6, J15.8, J15.9, J18.8, J 69.0, U69.01, J95.851, J85.1, J87.01 |
| Skin and soft tissue infections (SSTI) | L02.91, L02.92, L02.93, L03.0, L03.211, L03.213, L03.313, L03.11, L03.90, L03.90, L08.9, L72.0, M72.6, M79.9, N49.2, A46, R02.0, Y04.1XXA, Z87.2 |
