# Supplementary figures and images for: Evaluating Antibiotic Treatment Guideline Adherence to Ongoing Antibiotic Stewardship in a Tertiary Care Setting: A Retrospective Observational Study
Source: Can J Infect Dis Med Microbiol. 2024 Apr 17;2024:6663119. doi: 10.1155/2024/6663119 (PMC11042908; doi:10.1155/2024/6663119)

**Supplementary Table 2:** Hospital Antibiotic Treatment Guideline


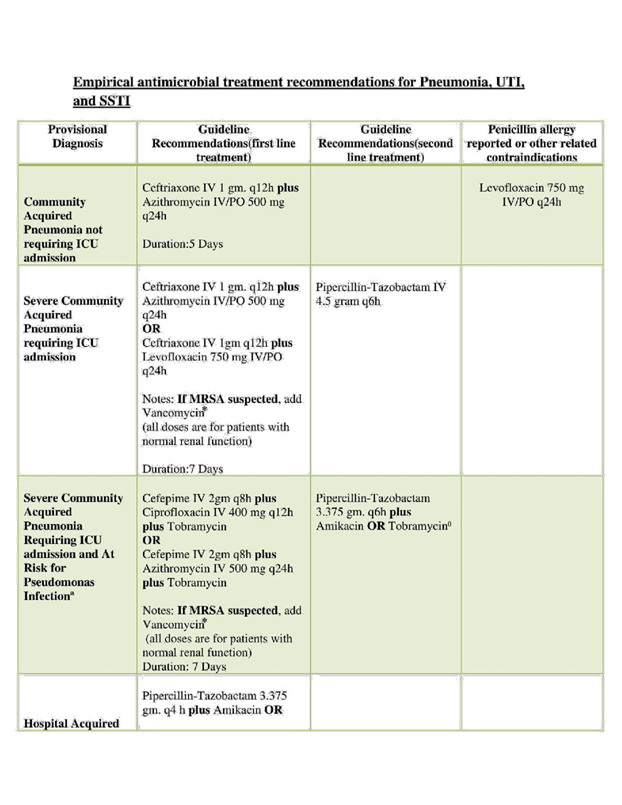


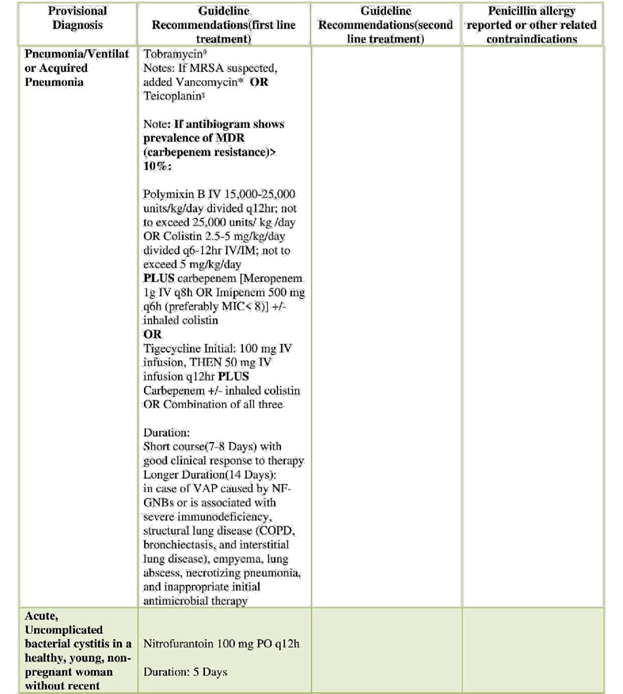


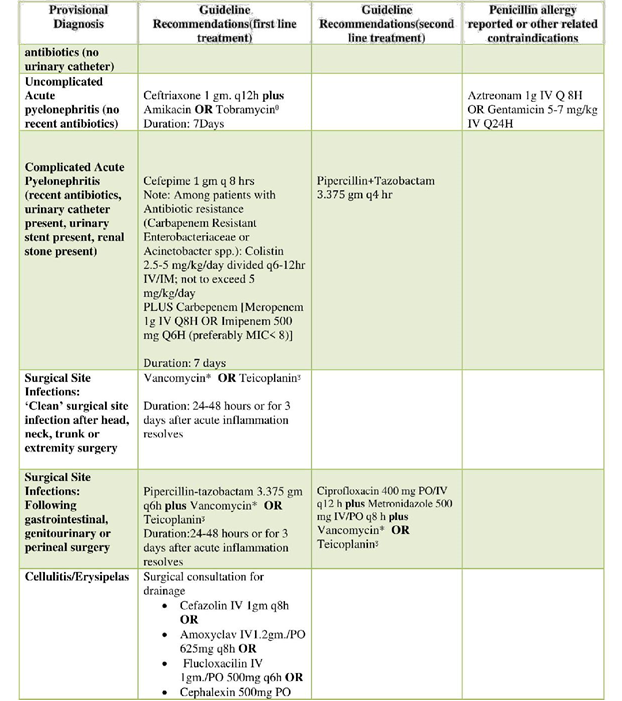


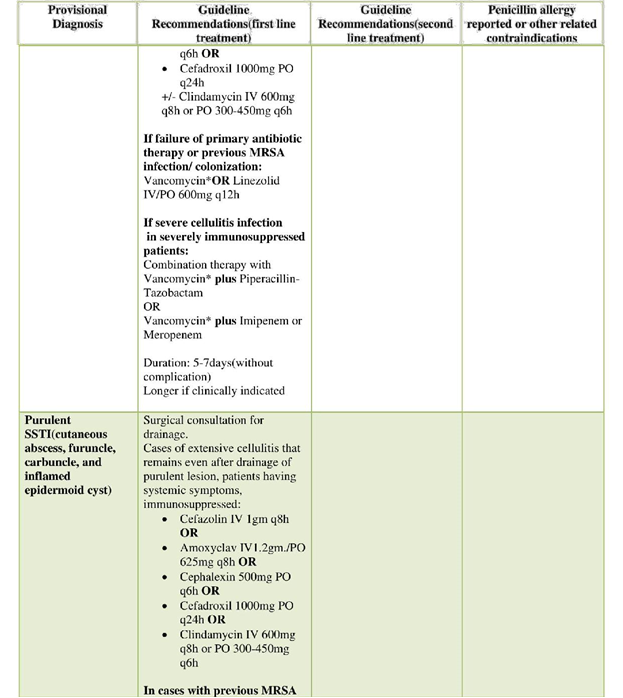


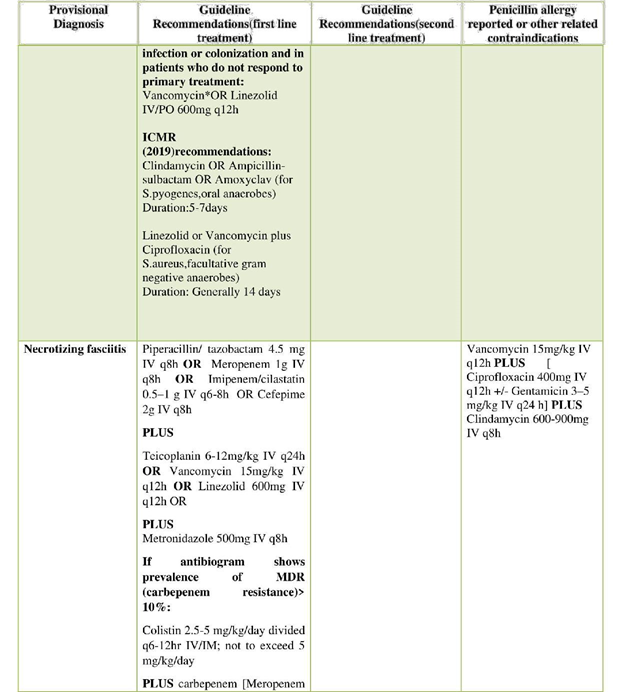


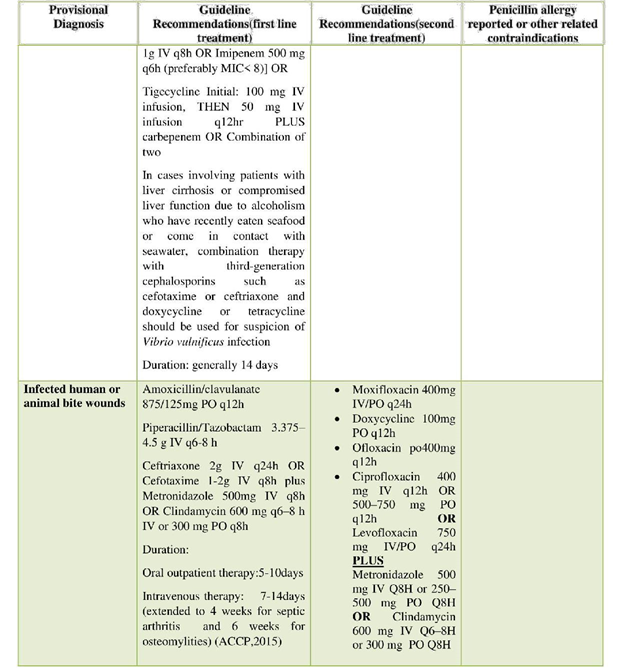


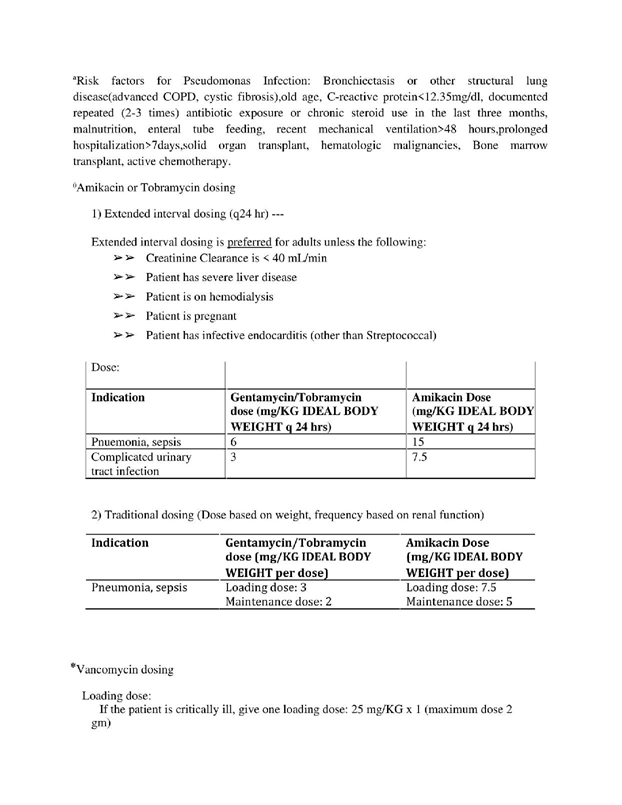


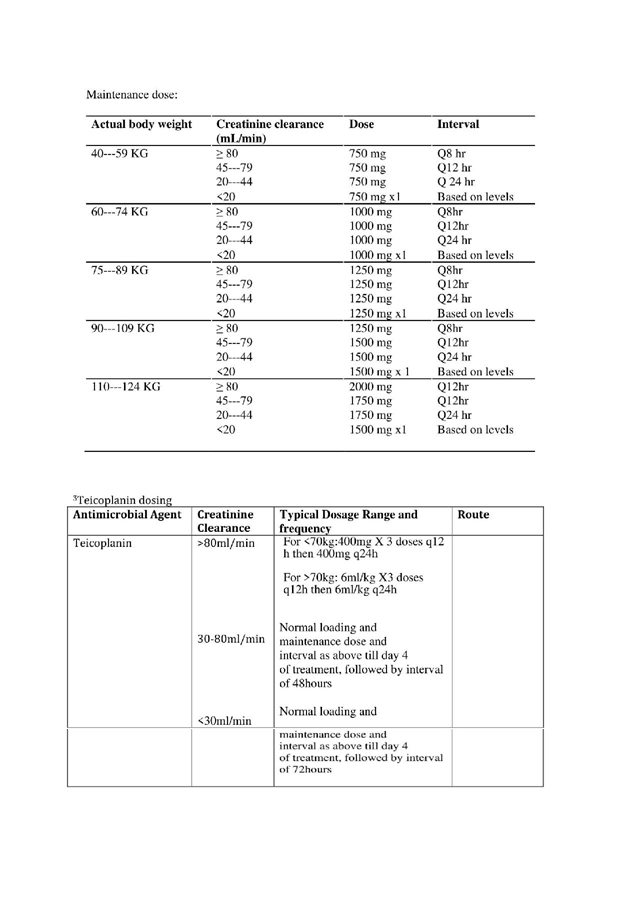

Supplement: Supplementary Materials — Supplementary Table 1: infectious disease diagnosis of patients and ICD-10 categories. Supplementary Table 2: hospital's antibiotic treatment guideline. [file 6663119.f1.zip › Supplementary Table 2_26March 2024.docx]
